# Textronic Capacitive Sensor with an RFID Interface

**DOI:** 10.3390/s24123706

**Published:** 2024-06-07

**Authors:** Patryk Pyt, Kacper Skrobacz, Piotr Jankowski-Mihułowicz, Mariusz Węglarski

**Affiliations:** Department of Electronic and Telecommunications Systems, Rzeszów University of Technology, ul. Wincentego Pola 2, 35-959 Rzeszów, Poland; k.skrobacz@prz.edu.pl (K.S.); wmar@prz.edu.pl (M.W.)

**Keywords:** RFID, UHF, capacity measurement, capacitive sensors, active identifier, semi-passive identifier, textronic, textronic sensors, textronic RFID identifier

## Abstract

This article presents an innovative combination of textile electrical circuits with advanced capabilities of electronic RFID sensors, indicating the revolutionary nature of the development of textronics, which is used in various areas of life, from fashion to medicine. A review of the literature relating to the construction of textronic RFID identifiers and capacitive textronic sensors is performed. Various approaches to measuring capacity using RFID tags are discussed. This article focuses on presenting the concept of a capacitive sensor with an RFID interface, consisting of a microelectronic part and a textile part. The textile part is based on the WL4007 material, where antennas and capacitive sensors are embroidered using SPARKFUN DEV 11791 conductive thread. The antenna is a half-wave dipole designed to operate at a frequency of 860 MHZ. The microelectronic part is sewn to the textile part and consists of a microcontroller, an RFID-integrated circuit and a coupling loop, placed on the PCB. The embroidered antenna is coupled with a loop on the microelectronic module. This article focuses on presenting various designs of textronic electrodes, enabling various types of measurements. Article presents capacitance measurements of individual sensor electrodes, made using a measuring bridge and a built RFID tag. The sensors’ capacity measurement results are shown.

## 1. Introduction

In today’s world, the development of intelligent textile technologies (textronics) is revolutionizing many areas of life. The literature describes the use of intelligent textile technologies in areas such as fashion, medicine, the military, and sports. Authors have emphasized the evolution of these technologies and their increasing functionality, including the ability to detect and respond to changing environmental conditions. The literature on the subject contains various applications of textronics, including sensors, electrodes, energy storage systems, and many others [1]. Textronics research is focused on three main levels of integration of electronic components: fabric-adapted, fabric-integrated, and fabric-based.

RFID technology, although initially used mainly in logistics and identification, is now used in various fields. This is the result of continuous development that has expanded the scope of functionality of RFID technology, enabling (among other things) responses to external stimuli and the generation and storage of energy and information [2,3].

The growing interest in textronics from both enterprises and researchers suggests prospects for the further development of this field in the future. The pursuit of synergies between textile materials and advanced technologies such as RFID opens up new opportunities to create intelligent, adaptive, and interactive systems that can significantly impact our everyday lives. Textronic capacitive sensors with an RFID interface are a pioneering combination of these two areas (textiles and electronics), combining innovative approaches to textile materials with the advanced capabilities of RFID technology.

These kinds of identifiers are made from textile materials in which conductive elements, such as thread, paste, or paint, are incorporated. These elements form an electric circuit, along with measuring electrodes. These electrodes have the ability to change their electrical parameters in response to changing environmental conditions, making them an ideal tool for sensory applications.

### 1.1. Textile Antennas

There are many methods for integrating antennas into textiles. In publication [4], the authors examine the electrical properties of dipole-type antennas made by embroidering with conductive threads on fabrics, as well as printed with silver ink on fabrics. They propose the optimization of dipole antennas intended for the implementation of radio frequency identification (RFID) tags. This study takes into account the influence of bending and thread density on the performance of embroidered antennas. The comparison between the silver thread and the ink shows that the parasitic capacitance of the conductive threads is much higher, but the structure of the silver conductive threads has a greater potential to improve the conductor performance than the silver ink at high frequencies. The most important conclusions are the need to take into account the thread density in order to achieve optimal resonance frequency and the possibility of replacing silver ink and solid metal with conductive threads in the applications of smart wearable devices.

In another publication, the research addressed the limitations of embroidered antennas, such as the higher resistance of conductive threads compared to metal traces, lower geometry resolution, and greater stretchability. Mathematical models and research results are presented that analyze the impact of these limitations on the performance of antennas, both for ultra-high frequencies (UHF) and high frequencies (HF) [5].

The usability of identifiers with textronic antennas and the integration of textronics with electronic systems are also important issues. In [6], the authors present three progressive designs of UHF-RFID textile antennas on surgical masks. In their research, the authors used simulations and measurements which confirmed the good fit of the antenna designs at the 868 MHz frequency. The study also conducted reliability tests such as bending and skin contact. Additionally, due to the functionality of the integrated circuit, the design can be adapted to different types of textile sensors for different application scenarios. The production method involved embroidering patterns onto surgical masks using an embroidery machine. Impedance and reflectance measurements for the embroidered designs confirmed their compliance with the simulations.

### 1.2. Textile Capacitors and Capacitive Sensors

Capacitive sensors are widely used in various fields, such as industry, medicine, and consumer electronics. They are used to measure humidity, position, presence, liquid level, and many other parameters. Such sensors are aesthetically pleasing, can be covered, and are resistant to dirt and moisture. Their small size, measurement precision, and the possibility of integration with RFID technology make them attractive in many applications [7,8]. Various technologies are used to produce textile capacitors and capacitive sensors, which combine traditional textile methods with innovative methods of incorporating conductive materials into these textiles.

The research in [9] on novel textile solid-state capacitors based on two-dimensional materials focuses on the use of graphene and boron nitride to create conductive and dielectric fabrics, respectively, which are then integrated to form a textile capacitor. The research includes the preparation of graphene- and h-BN-based inks, as well as the application of these inks on polyester fabrics using a dipping and drying process. The test results show that the obtained fabrics are characterized by appropriate electrical properties and hydrophobicity. Once these fabrics are integrated, a textile capacitor is created that has the ability to withstand repeated bending and washing. The results suggest the potential for using this type of capacitor in textile electronics.

Another method of producing textile circuits is to use silver-plated conductive yarn on a polyester fabric base. The design and application of an embroidered textile sensor have been discussed in the literature. The authors of [10] conducted research on the use of an embroidered textile sensor with a spiral capacitive structure to measure capacitance in response to sucrose solutions of various concentrations and different frequencies of the measurement signal. Their study showed that the capacity changes linearly with the change in sucrose concentration in a specific frequency range (250–500 Hz). Their experiments showed that the sensor capacity decreases with increasing sucrose concentration and frequency. The conclusions of this study confirm the feasibility of using an embroidered textile sensor to measure the concentration of sucrose solutions in a linear manner in a specific frequency range.

Adaptation of the weaving process enables full integration of the sensor in the fabric, which provides better repeatability compared to the previously discussed embroidered and printed sensors. The hypothesis put forward in the [9] concerns testing the feasibility of using weaving technology to fully integrate a capacitive sensor in the fabric and assessing its effectiveness in measuring humidity and presence detection. This sensor was designed for humidity measurement and presence detection. The sensor’s response to humidity was investigated by measuring the capacitance with an LCR meter in the range of 20 Hz to 20 kHz in an environmental chamber, varying from 30% to 90% relative humidity (RH), at a temperature of 20 °C. Occupancy response was then assessed by measuring the sensor capacitance in response to surface loading. The test results confirmed the functionality of the sensor as both a humidity sensor and a presence detector.

In addition to printing, embroidering and spinning textile circuits, scientists are working on electro-spinning and micro-enveloping methods. Using these techniques, a highly sensitive, durable and ultra-thin capacitive sensor based on a network structure made of fabric, called AFCS, was created in [11]. The hypothesis put forward in the analyzed article assumed that the new sensor would be characterized by high sensitivity and a super-thin dielectric layer, which would enable its use not only in textronics but also in skin electronics. The most important conclusions were the confirmation of the promising potential of AFCS in tracking respiratory activity, monitoring muscle activity and measuring fingertip pressure.

In addition to examining the possibilities of using various technologies and materials, various patterns and structures of capacitance sensors are tested. The authors of [12] investigate the influence of various structural parameters of the fabric on the mechanical and electrical properties of the sensor and its application in the detection of human movements. The hypothesis assumes that a 3D fabric with an appropriately selected structure can provide excellent mechanical and electrical properties, enabling effective detection of body movements. The research methods include designing the structure of a flat knit fabric, examining its mechanical and electrical properties, and testing the sensor to detect different types of pressure, such as finger pressure, surface pressure, and punch force. The study results confirm that the sensor can be effectively used to monitor various body movements, indicating its promising application in flexible and wearable devices. This study used a spatial flat-knitted fabric and materials such as polyamide and polyester, as well as electrodes made of polyester fabric with a nickel-copper coating.

### 1.3. RFID Textronic Sensors

The three levels of electronics integration in textiles also apply to electronic capacitive sensors. Such sensors may be integrated circuits sewn into the material, they may be incorporated into the material by sewing with conductive threads, sputtering conductive layers, or printing with conductive paint, or the appropriately manufactured textile material itself may be a capacitive sensor. For example, [8] presents a passive UHF RFID system with a capacitive sensor interface with low energy consumption. The design of a base station and a passive RFID tag is described, which consists of an inductively powered meander dipole antenna and an integrated circuit (IC) containing remote power supply, communication and sensor interface circuits. A highly efficient differential rectifier with self-compensating thresholds is used. A low-current phased loop (PLL)-based sensor interface was used to differentially read the capacitive sensor on the tag. Data from the sensor’s modulated PWM signal were sent to the base station via backscatter. The tag chip was implemented in a 0.18 µm CMOS process. The most important conclusions are the linear response of the system to changes in the sensor capacitance value and the effectiveness of receiving data from the tag by the base station.

Another approach is to measure capacitance by recording changes in the parameters of an embroidered antenna, which is also a sensor. The authors of [13] propose an innovative method for detecting the parameters of RFID sensors based on the non-linear variability of the impedance of an integrated circuit depending on the level of transmitted power. The authors present a hypothesis regarding the use of this method to detect the parameters of RFID sensors and research methods, including both RCS measurements of tags and computer simulations. The results of experiments and simulations confirm the non-linear variability of IC impedance with the level of transmitted power, which allows the use of this feature for detecting tags in various measurement conditions. The conclusions suggest that the proposed method is a promising way to solve problems related to the detection of RFID sensors in various environments [13].

This article presents an innovative capacitive sensor with an RFID interface. The main part of the sensor is a microelectronic module made on a PCB, sewn to the textile material. The sensor antenna was sewn using conductive thread on the material. This antenna couples to the loop on the microelectronic module. The operation of the RFID interface was tested, the identifier memory was read and written via the radio interface. Details regarding the coupling of the antenna module and the microelectronic module can be found in [14,15,16], as they are not the subject of this publication.

The three electrode patterns of capacitive sensors were sewn with conductive thread onto a textile material. The capacitance of electrodes was measured using the bridge method. The electrodes were stitched onto the microelectronic module using a conductive thread, and then the electrode capacitance measurements were read, provided by each capacitive sensor. This article focuses on showing the possibilities of using this type of sensor in smart clothes. As an application example, the registration of changes in electrode capacitance under the influence of an object approaching the electrodes was tested. In this way, touch sensors integrated with clothing can be implemented.

## 2. Construction of a Capacitive Textronic Sensor with an RFID Interface

**Figure 1:**
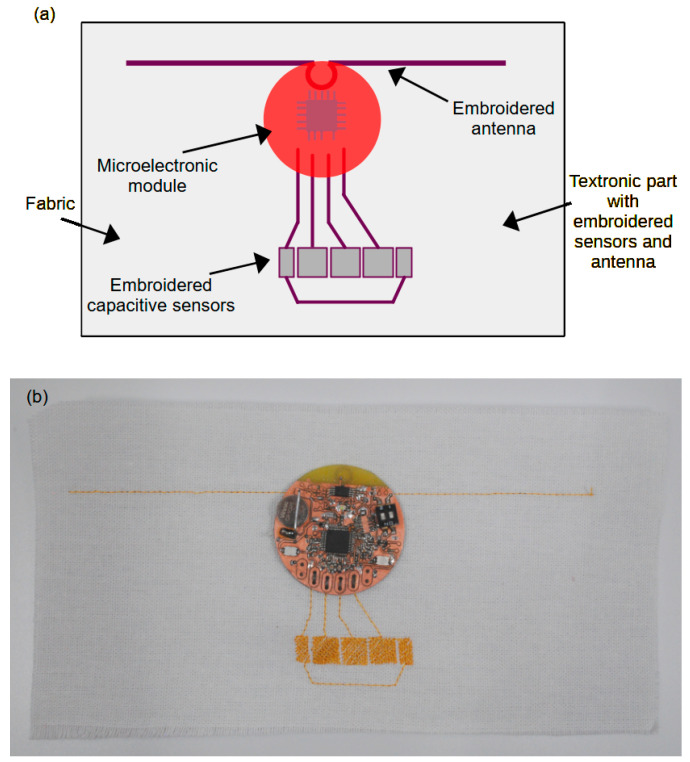
Demonstrator of a textile capacitive sensor with an RFID interface: (**a**) symbolic representation of the individual components of the demonstrator; (**b**) photo of the completed demonstrator.

This article presents the concept of a capacitive sensor with an RFID interface, which consists of a microelectronic part and a textile part, as symbolically shown in Figure 1a. Figure 1b also shows a photo of the device. The microelectronic part is made in the form of an electronic circuit on a PCB, while the textronic part is made from fabric constituting the base, on which the RFID identifier antenna and capacitive sensors are sewn with conductive threads. The microelectronic module is sewn to the textile module with the same conductive threads that create the sensors. The stitched antenna is inductively coupled to a coil made from a copper track on the PCB.

### 2.1. Microelectronic Module

**Figure 2:**
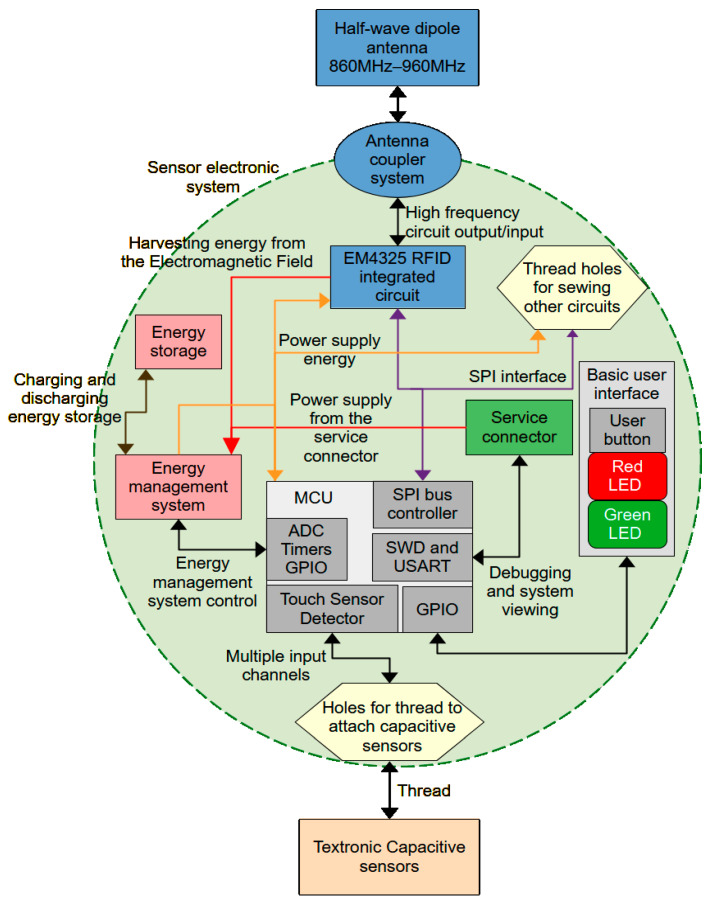
Block diagram of a textronic capacitive sensor with an RFID interface.

The concept of the microelectronic module in the form of a block diagram is shown in Figure 2. The module demonstrator was created on a double-sided PCB laminate in which the circuit paths were milled. There are no obstacles to applying the demonstrator on a different surface, for example on flexible, copper-plated kapton. In this case, the system will be more flexible and, above all, thinner. The demonstrator is relatively large and has a diameter of 3 cm, but if the elements were used in smaller housings, the diameter of the demonstrator could be reduced to the size of a button. Then, the button in the example application could be sewn to a shirt with a conductive thread and the antenna could be inductively coupled.

The central part of the device is the STM32L053C8T6 microcontroller produced by STMicroelectronics, which communicates with the EM4325 RFID system produced by EM Microelectronic, via the SPI bus. Depending on the purpose of the device, the microcontroller can play the role of a master or slave system. Via the SPI interface, it is possible to configure the EM4325 system and bi-directionally transmit information between the microcontroller and the reader–programmer communicating with the RFID tag. The SPI bus lines are also connected to the thread holes, which makes it possible to attach another module located on the same material to the microelectronic module, which has the option of communicating using SPI. Such an additional module can perform any function, such as a display, an additional set of sensors, etc.

The EM4325 is a three-class, two-generation RFID integrated circuit compliant with ISO/IEC 18000-63 [17], ISO/IEC 18000-64 [18], and EPC Class-1 Generation-2 [19]. It operates in the UHF frequency range from 860 MHz to 960 MHz. The RF output of this system is connected to a loop made from a copper track on the PCB. This loop is coupled to the embroidered antenna, and both elements are isolated from each other. Details of the coupling system of the microelectronic module and the textile antenna can be found in [13,14,15,16]. This solution has many advantages over the solution of sewing the antenna to the microelectronic module. The first most important advantage is the permanent connection of the antenna with the module, which is not affected by the use of the device, during which the sewn connections weaken as a result of use. The second most important advantage is the stability of parameters, as the coupling of the antenna and coil on the module does not change due to wear of the system. The third most important advantage is the ability to modify the shape of the embroidered antenna and adapt it to the microelectronic module and the product on which the identifier is to be placed [20,21,22].

**Figure 3:**
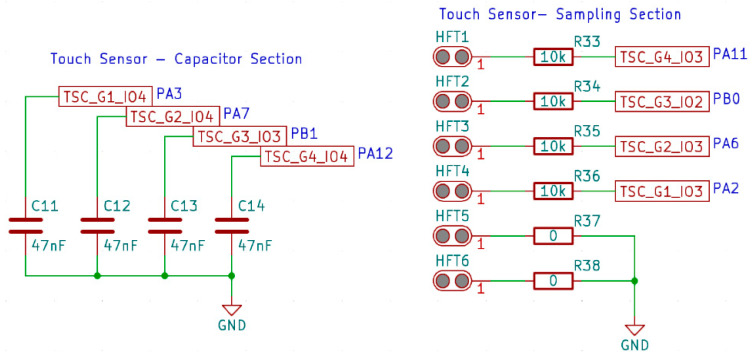
Diagram of the electrical connection of capacitive sensors with the channels of the TSD and the method of connecting sampling capacitors.

The used microcontroller is equipped with a TSD (touch sensor detector) peripheral system, dedicated to operating capacitive buttons. This system uses the surface charge transfer method to detect changes in the capacitance of the copper traces connected to the appropriate pins of the microcontroller. In the case of the microelectronic module presented here, the channels of the TSD system are connected to thread holes through which the PCB is sewn to the material, using the same threads that create the capacitive sensor. To summarize, capacitive sensors are attached to the input channels of the TSD system, creating an electrical connection, the diagram of which is shown in Figure 3 [23].

The elements in Figure 3 marked as HFT1-6 are holes for the conductive thread. These holes are covered with copper, which ensures the electrical contact of the microelectronic module with the conductive thread. Holes HFT5 and HFT6 are used to connect the common mass electrode of the sensor, while the remaining holes are used to connect measurement electrodes to individual TSD channels. The input channels are divided into groups. In Figure 3, the input channels are marked as TSC_GX_IOY, where X is the group number and Y is the channel number in a given group. Measurement in the same group can be performed sequentially on subsequent sensors. The use of several analog groups of input-output ports in the presented demonstrator enables the simultaneous measurement of several sensors at the same time. One port in each group is dedicated to the sampling capacitor C11–C14. Only one such capacitor can be connected to a given group of ports. The remaining ports in each group are dedicated to connecting sensor electrodes.

The surface charge transfer acquisition method was used in the RFID tag to measure the capacity of the sensor. This method allows one to measure the capacity of a capacitor composed of one measuring electrode and a reference electrode. The surface charge collection method consists of charging the capacitance of the sensor electrode *C*x and transferring part of the charge accumulated there to the sampling capacitor *C*s. This sequence is repeated until the voltage on the sampling capacitor *C*s reaches the set threshold *V*_IH_. The number of charge transfers required to achieve a given sample is directly proportional to the capacitance of the sensor electrode. When the capacitance of the sensor changes, the number of cycles needed to charge the capacitor to the appropriate level also changes [23,24].

A sewn connection changes its parameters over time. The impedance of the connection changes as the thread loosens or tightens and the thread itself deteriorates. However, in this case (unlike the connection between the microelectronic module and the antenna), the capacitive sensor can be calibrated programmatically and the impact of changing the connection parameters of the embroidered sensor with the microelectronic module can be eliminated [24].

The sensor is powered by the battery through the energy management system. This system distributes and adjusts the power parameters to the microcontroller and RFID system. The energy management system is controlled by a microcontroller that constantly monitors the power supply parameters. The battery can be recharged with energy obtained from the electromagnetic field of the reader–programmer or through the service connector.

The service connector allows one to connect a microelectronic programmer to the module, with which the microcontroller can be programmed. Through this connector, it is possible to monitor the operation of the identifier and power the identifier. There is also a button and two LEDs on the module that provide basic communication with the user.

### 2.2. Textile Part

**Figure 4:**
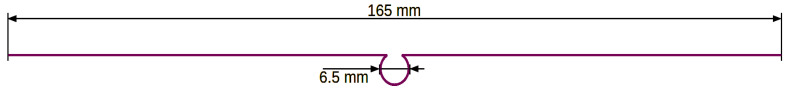
Design of a textronic antenna, which is a half-wave dipole operating in the UHF frequency range.

This section presents designs and recommendations regarding the shape of textronic sensors and antennae made on flat surfaces. The basis of the textile part is a material with the trade name WL4007. This material is 0.8 mm thick and its electrical permittivity ε_r_ is 2 F/m. The antenna and capacitive sensors are embroidered using a conductive thread with the trade name SPARKFUN DEV 11791 [14]. The antenna is a half-wave dipole made of a single strand. In the central part of the dipole, there is one loop constituting the coupling system between the antenna and the microcontroller system. Figure 4 shows the antenna design.

Individual electrodes of the sensors were embroidered in the form of fields using conductive thread. This field acts as a capacitor that is alternately charged and discharged. The capacitance of this capacitor depends on the shape of the field and the electrical permittivity of the dielectric surrounding it. The capacitance is proportional to the electrode area.

The capacitance of the measuring electrode consists of parasitic capacitance, self-capacitance, and capacitance associated with the detected object. Parasitic capacitances are capacitances related to the paths leading from the electrodes to the measurement system, capacitances related to the presence of adjacent measurement electrodes, etc. The self-capacitance of the measuring electrode is the capacity resulting from the electric field between the measuring electrode and the ground electrode.

The capacitance associated with the detected object is proportional to the part of the surface area of the detected object that corresponds to the part of the surface of the measuring electrode. Therefore, the surface area of the sensor’s measuring electrode should be similar to the surface area of the detected object. Increasing the surface area of the measuring electrode above the surface area of the detected object will not increase the capacitance associated with the detected object, but it may increase parasitic capacitances. Additionally, the system could react to objects that it should not detect and which are too close to the too large measuring electrode.

It is recommended to design sensors of the same size as the object being detected. The sensor should be at least four times wider than the thickness of any dielectric covering the sensor [24].

**Figure 5:**
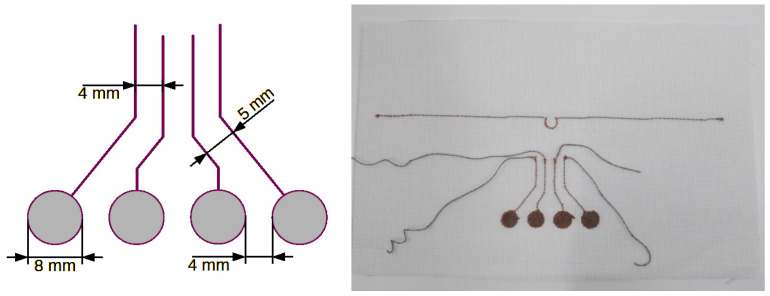
Textronic capacitive sensor in the shape of circles.

**Figure 6:**
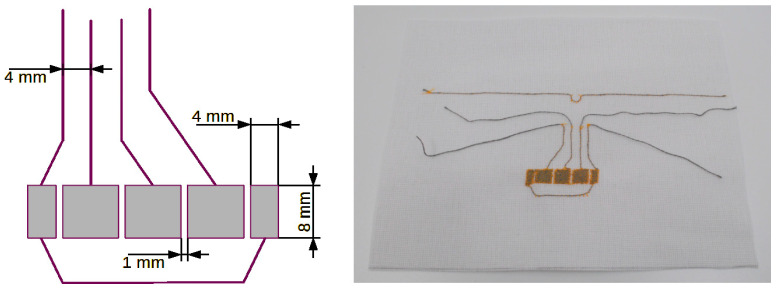
Textronic capacitive sensor in the shape of a slider, enabling the detection of linear movement of objects.

**Figure 7:**
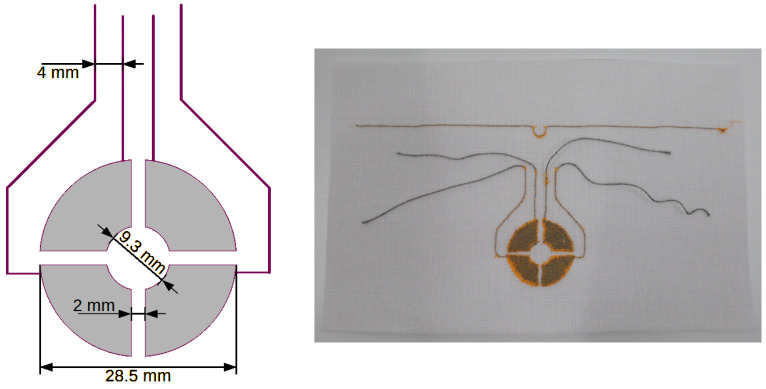
Textronic capacitive sensor in the shape of ring fragments arranged around a common axis. The sensor enables the detection of the rotational movement of an object.

**Figure 8:**
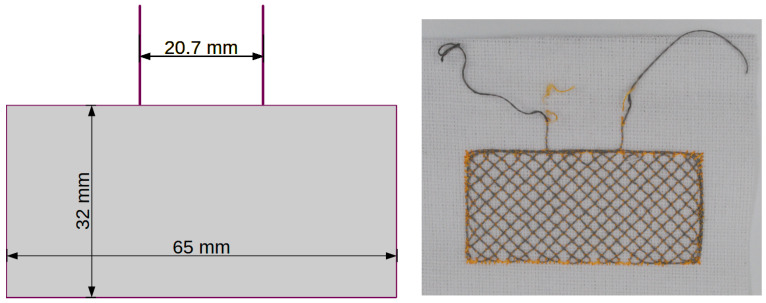
Ground electrode, placed under the measuring electrodes.

**Figure 9:**
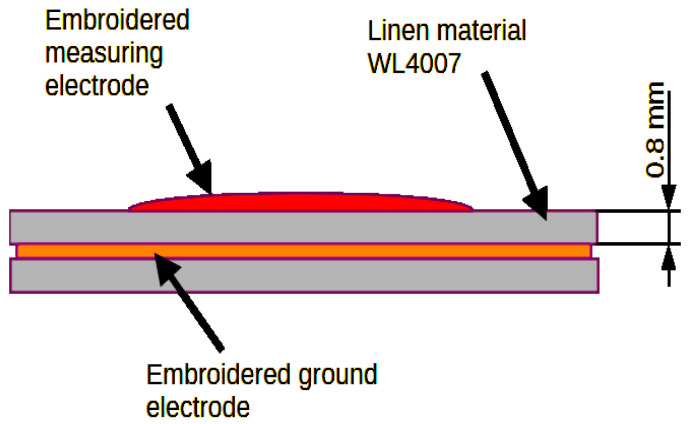
Cross-section of an embroidered capacitive sensor.

Figure 5, Figure 6, Figure 7 and Figure 8 show designs and photos of three different designs of measuring electrodes and a ground electrode, which are part of the capacitive sensor. Capacitive sensors consist of two layers placed in close proximity, one on top of the other. The top layer is a measuring electrode and the bottom layer is a ground electrode (Figure 9). These electrodes are separated by a dielectric, which in this case is linen material WL4007. An important feature of the structure is the distance between the electrodes, equal to the base WL4007 material’s thickness of 8 mm, and the shape of these electrodes, which can take various forms, such as plates, cylinders or meshes. The shape and distance between the electrodes have a significant impact on the sensor’s capacity.

The first pattern (Figure 5) consists of four circles that can be used as touch buttons. Each circle is connected to one input channel of the TSD system. The electrode can have any shape, but simple shapes are preferred: circles, squares, rectangles and ovals. In the presented sensor, individual electrodes have the same shape and size and are symmetrically sewn on the fabric, which gives them similar parameters.

The second design (Figure 6) is a linear (also called slider) sensor, which is a set of adjacent capacitive electrodes arranged on one common axis. The number of electrodes depends on the required size and resolution of the sensor. The four individual electrodes are connected to the four input channels of the TSD system. The start and end electrodes are connected to one channel, which allows the sensor to be larger while limiting the number of channels used. The outer electrodes are half the size of the middle electrodes, so the electrical capacity of all electrodes is similar. Such a sensor can detect objects by moving along the sensor.

The last pattern (Figure 7) has the form of four ring fragments. This is a so-called rotation sensor that allows one to detect changes in the position of an object around the central axis. Each ring is connected to one channel of the TSD system. Increasing the number of ring fragments (the number of sensor electrodes) results in an increase in resolution. Each electrode is connected to one channel. The measurement of channels must be simultaneous because an object located near one electrode also affects the neighboring ones. Simultaneous measurement of all channels allows one to avoid the incorrect interpretation of the results.

In addition to the measuring electrodes, a ground electrode is also embroidered (Figure 8), which should be placed under the measuring electrodes. The electric field lines close between the measuring and ground electrodes. These lines may be disturbed by an object or a change in dielectric parameters. The potential at the ground electrode is the reference potential of the measurement system. The ground electrode also serves as a screen that reduces the undesirable influence of the environment outside the measurement area on the sensor’s capacity.

In the rest state of the sensor, the field lines close between the measuring electrode and the ground electrode. The capacitance of such a system is influenced by the shape of the electrodes and the permittivity of the material through which the field lines pass. Changing the ambient parameters (for example, due to humidity, composition of matter, etc.), and thus changing the electrical permittivity, affects the capacity of the system, which is registered by TSD. Also, the appearance of another object near to electrodes that changes the distribution of electric field lines changes the capacitance of the system, which makes it possible to record indirect touches of the electrodes or move objects along and around the axis of the presented sensors. Differences between individual capacitive sensors can be compensated in software or hardware. The most accurate result can be obtained when the individual electrodes of capacitive sensors have similar capacity. Therefore, it is recommended to use sensors of the same shape in a given application. Embroidered sensors can have a shape optimized for capacity, but their appearance can vary if part of the pattern is sewn with non-conductive thread.

## 3. Software Algorithm

**Figure 10:**
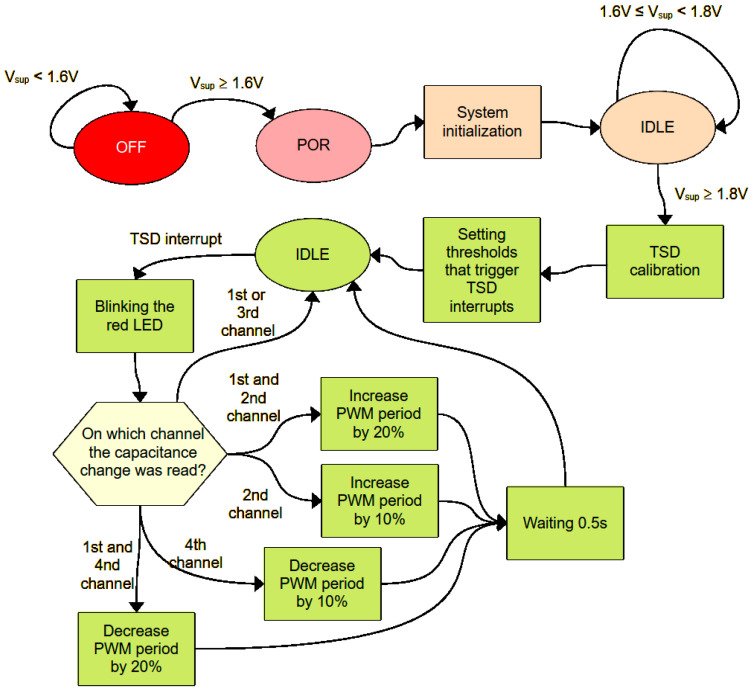
Operating algorithm of the demonstration program of a capacitive textile sensor with an RFID interface.

The software algorithm managing the operation of the textronic capacity sensor performs basic functions, demonstrating the possibilities of using it to monitor the parameters of the marked object. The block diagram of the program’s operation algorithm is shown in Figure 10. If the supply voltage is less than 1.6 V, the system is turned off. When the voltage increases to 1.6 V, the system starts and power-on reset (POR) is performed, which ensures the correct initialization of the microcontroller registers during startup. Then, the user program is launched, in the initial stage of which peripheral systems such as a TSD (touch sensor detector), SPI and USART interface, counters, analog-to-digital converters, input–output ports, and DMA (direct memory access) are initialized. As a result of initialization, one of the counters starts to generate a PWM signal with a *T*_TPWM_ period of 3 s and a high state duration of 0.3 s, which controls the green LED. After initialization, the microcontroller goes into sleep mode, waiting for the supply voltage to reach 1.8 V. After reaching the required voltage, the capacitance of all four capacitive sensors is measured. The measured value is the *C*_mrv_ reference value to which subsequent capacity measurements are compared. Then, the lower and upper thresholds are set to activate the interrupt from the TSD system. These thresholds are 80% and 120% of the *C*_mrv_ value, respectively [25].

The microcontroller then goes to sleep. If the sensor capacity changes by 20%, an interrupt is triggered and the red LED lights up for 0.3 s. In the interrupt handling procedure, a decision is made based on the channel number on which the capacity change was recorded. Activating only the first or third channel has no effect. Simultaneous activation of the first and second channels increases the period of the *T*_TPWM_ signal driving the green LED by 20%, while activating only channel 2 increases this period by 10%. In turn, activating the first and fourth channels simultaneously reduces the *T*_TPWM_ period by 20%, and activating only the fourth channel reduces this period by 10%. These changes refer to the initial value of the *T*_TPWM_ signal period, which is 3 s. After changing the PWM signal period and, at the same time, changing the blinking frequency of the green LED, the microcontroller waits 0.5 s and then goes into sleep mode again [26].

## 4. Verification of Correct Sensor Operation

**Figure 11:**
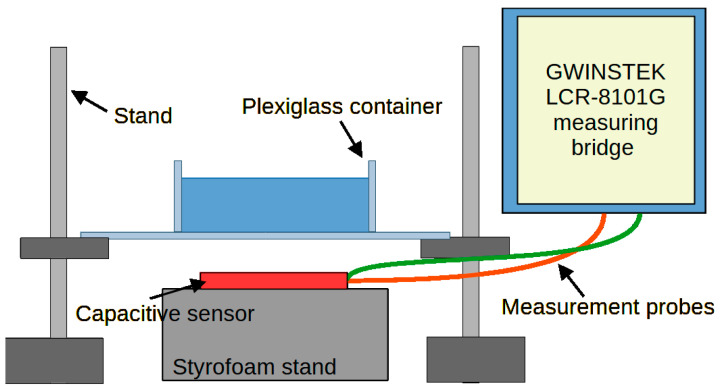
A measuring system used to determine the capacity of a capacitive sensor, depending on the distance of the object located in front of the sensor. The red and green lines symbolize the measurement probes, attached to the mass electrode and the measuring electrode of the textronic sensor, respectively.

**Figure 12:**
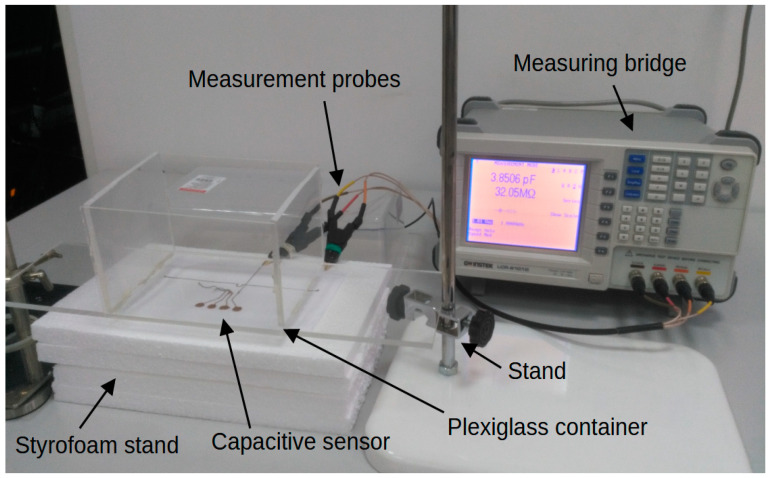
Photo of the measuring system used to measure the capacity of the sensor depending on the distance of the object.

**Figure 13:**
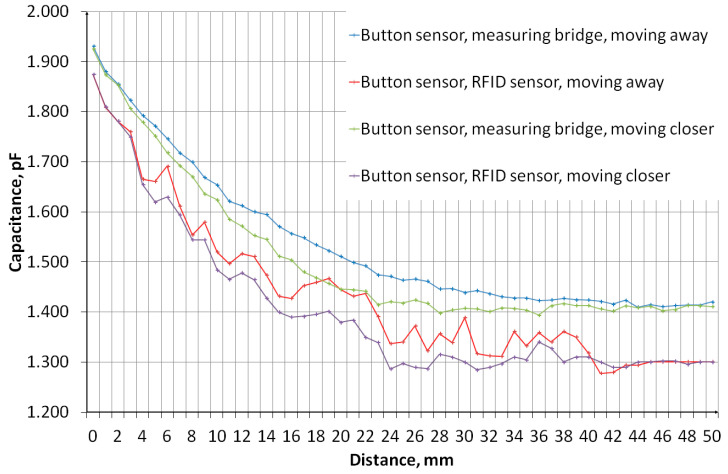
A plot showing the results of capacitance measurements of a button-shaped electrode sensor for various object distances.

**Figure 14:**
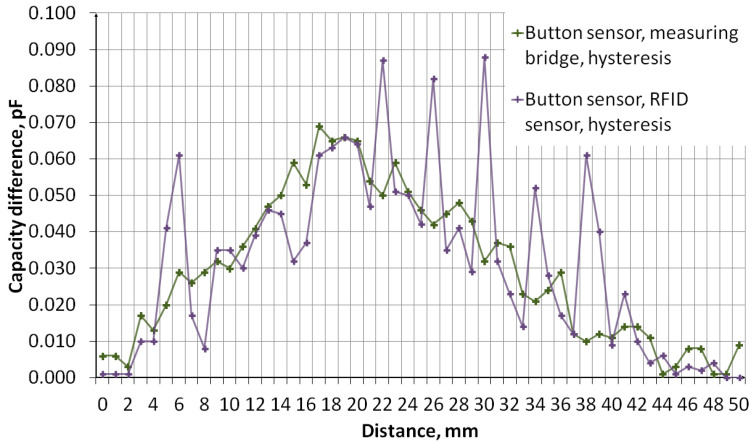
A plot showing the hysteresis of a button-shaped capacitive sensor, reflecting the difference in the sensor’s capacitance as the object approaches and moves away.

**Figure 15:**
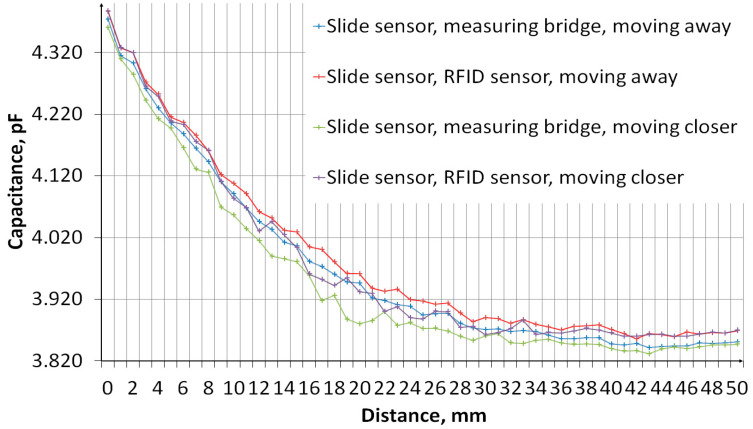
A plot showing the results of capacitance measurements of a sensor with a slider-shaped electrode for various object distances.

**Figure 16:**
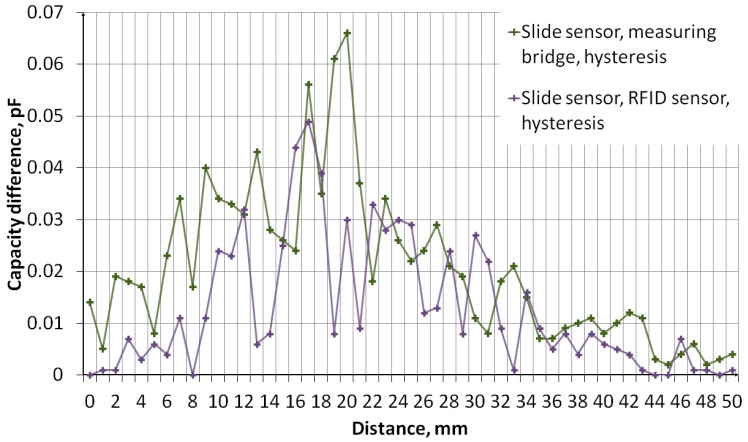
A plot showing the hysteresis of a slide-shaped capacitive sensor, reflecting the difference in the sensor’s capacitance as the object approaches and moves away.

**Figure 17:**
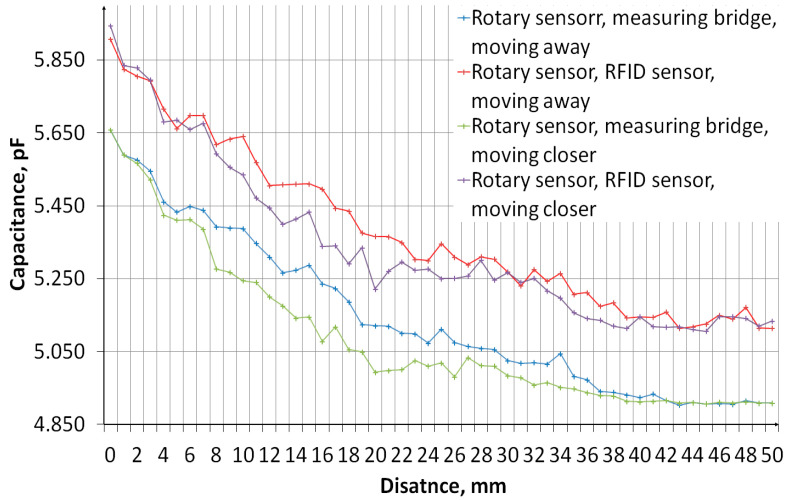
A plot showing the results of capacitance measurements of a sensor with an electrode in the shape of a rotary sensor for various object distances.

**Figure 18:**
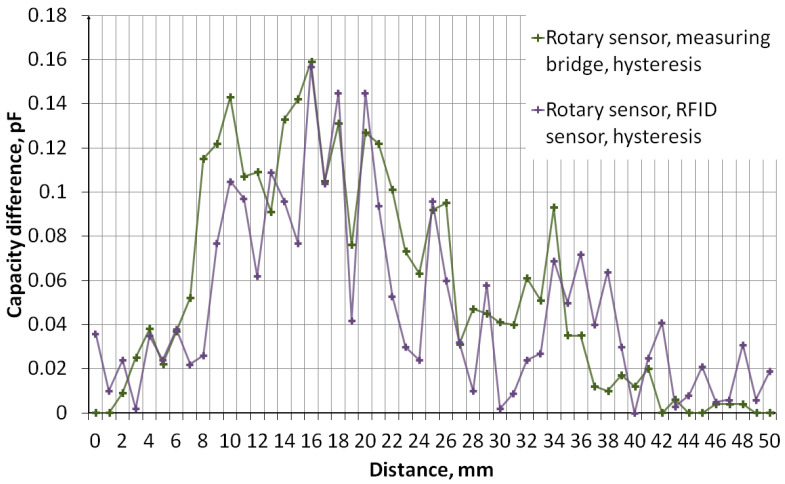
A plot showing the hysteresis of a rotary-shaped capacitive sensor, reflecting the difference in the sensor’s capacitance as the object approaches and moves away.

The measurement of sensors’ capacity depending on the distance of the object was performed. The measurement system, the diagram of which is shown in Figure 11, consisted of an embroidered capacitive sensor placed on a styrofoam base. Above the sensor, there is a container made of plexiglass with a wall thickness of 2 mm. The container rests on stands, which makes it possible to adjust the distance of the container bottom from the sensor. The object was first moved away, from 0 mm to 50 mm, then the object was moved closer, from 50 mm to 0 mm. The container was filled with water. Figure 12 shows a photo of the stand. The capacity was measured using a GWINSTEK LCR-8101G measuring bridge and an RFID identifier. When measuring with a bridge, the measurement signal had an amplitude of 1 V and a frequency of 1 MHz. The measurement was made for a model of series connection of capacitance and resistance. The results are presented in the form of graphs in Figure 13, Figure 14, Figure 15, Figure 16, Figure 17 and Figure 18, respectively, for the button-shaped electrode, the slider electrode, and the rotary sensor electrode.

Hysteresis is visible in the plot reflecting the dependence of the sensor capacity on the distance of the detected object. The difference in capacitance between moving the object closer and further away for a given distance was calculated to quantify the sensor hysteresis using Formula (1):

(1) $$C_{diff}(d)=\left|C_{meas1}(d)-C_{meas2}(d)\right|,$$

where *C*_diff_ is the capacity difference, *C*_meas1_ is the capacity measured while the object is moving away, *C*_meas2_ is the capacitance measured while the object is moving closer, and *d* is the distance between the sensor and the object.

**Figure 19:**
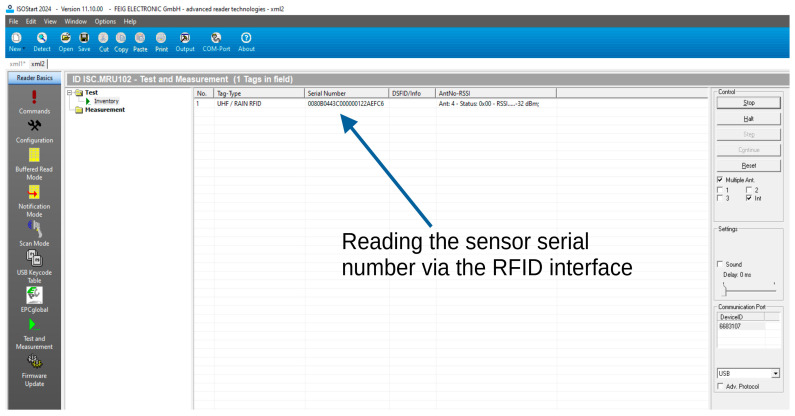
ISOStart program window showing the reading of the serial number of the sensor identifier via the RFID interface.

A communication test was performed between the identifier and the Feig ID MRU102 RFID reader, produced by FEIG Electronic. Figure 19 shows the ISOStart 2024 program, version 11.10.00, produced by FEIG Electronic. The program window shows the successful reading of the identifier serial number.

## 5. Conclusions

This article presents an innovative approach to the integration of textile smart technologies with RFID technology by creating a textronic capacitive sensor with an RFID interface. The proposed electronic identifier consists of a microelectronic module made on PCB sewn onto fabric, onto which the RFID antenna and capacitive sensors are embroidered using a conductive thread. The microelectronic module communicates with the RFID system and manages the operation of the sensors. This article also discusses the software algorithm managing the sensor’s operation, which allows for monitoring the parameters of marked objects.

The use of embroidery technology to create an antenna and sensor electrodes, then sew on a microelectronic module, opens the door to a number of potential applications in the field of smart clothes, health, or monitoring environmental conditions.

The results of verifying the correctness of the capacitance measurement of the embroidered electrodes by comparing the results of the RFID sensor readings with the values obtained by the bridge method confirm that the RFID sensors can be used successfully in textronic applications. The design of the sensor, namely the coupling of the embroidered antenna with the microelectronic module, allows for correct radio communication with the RFID reader and programmer.

Therefore, the results presented in this paper constitute an important contribution to the field of RFID sensor development, highlighting their potential in the field of capacitive measurements. At the same time, they suggest a promising path for further research and the implementation of this technology in practice.

## Data Availability

All calculated and measured data will be provided upon request to the correspondent authors by email with appropriate justification.
